# A Surface Plasmon Resonance Sensor for the Detection of Deoxynivalenol Using a Molecularly Imprinted Polymer

**DOI:** 10.3390/s110908654

**Published:** 2011-09-05

**Authors:** Sung-Wook Choi, Hyun-Joo Chang, Nari Lee, Hyang Sook Chun

**Affiliations:** Food Safety Research Center, Korea Food Research Institute, Sungnam 463-746, South Korea; E-Mails: swchoi@kfri.re.kr (S.-W.C.); hjchang@kfri.re.kr (H.-J.C.); nari@kfri.re.kr (N.L.)

**Keywords:** deoxynivalenol, polypyrrole, molecular imprinting polymer, surface plasmon resonance, synthetic receptor

## Abstract

The aim of the present work was to investigate the feasibility of applying the molecular imprinting polymer technique to the detection of the mycotoxin deoxynivalenol (DON) using a surface plasmon resonance (SPR) transducer. A molecularly imprinted polypyrrole (MIPPy) film was prepared via electropolymerization of pyrrole onto a bare Au chip in the presence of a template DON molecule. Atomic force microscope SPR analysis showed that the MIPPy film was deposited homogeneously on the Au surface, with a thickness of 5 nm. The MIPPy–SPR sensor exhibited a linear response for the detection of DON in the range of 0.1–100 ng/mL (R^2^ = 0.988). The selectivity efficiency of the MIPPy film for DON and its acetylated analogs 3-ADON and 15-ADON was 100, 19, and 44%, respectively. The limit of detection for DON with the MIPPy–SPR for a standard solution was estimated at >1 ng/mL. These results suggest that the combination of SPR sensing with a MIPPy film as a synthetic receptor can be used to detect DON.

## 1. Introduction

Deoxynivalenol (3α,7α,15-trihydroxy-12,13-epoxytrichothec-9-en-8-one, DON) is one of the trichothecene mycotoxins, chemically characterized by a common tetracyclic 12,13-epoxytrichothec-9-ene skeleton. DON is a secondary fungal metabolite produced by various species of *Fusarium*, especially *Fusarium graminearum* (*Gibberella zeae*) and *Fusarium culmorum*, both of which are important plant pathogens commonly found in cereals and other crops [1]. DON is very stable during the storage and milling of cereals or during the processing or cooking of food, and does not degrade at high temperatures [2,3].

Although DON is not among the most acutely toxic trichothecenes, the frequent occurrence of DON in cereals and cereal products represents a potentially serious human and animal health safety issue [4]. Consumption of foods and feeds contaminated with DON is associated with a variety of adverse health effects, including diarrhea and emesis [5,6]. To reduce the intake of DON, at least 37 countries have established or proposed the regulatory limits or guidance levels for DON in foods and feeds [7,8]. The guidance level for cereal and finished cereal products intended for human consumption was established at 100 μg kg^−1^ to 2,000 μg kg^−1^ [7,8]. Although these limits are set to protect the supply and trade of agricultural products, especially grains, the development of rapid, reliable, and sensitive analytical methods for the determination of DON is necessary.

To monitor DON contamination in foods or feeds, many researchers have performed studies using conventional analytical chromatographic methods, such as thin layer chromatography (TLC), high-performance liquid chromatography (HPLC) and gas chromatography (GC) [9–15]. Recently, immunosensors that use the antigen-antibody reaction have also been used for the rapid and easy determination of DON [16,17]. Nevertheless, these methods are expensive and affected by physical and chemical conditions. In addition, the development of toxin-specific immunoglobulins is difficult, as it typically involves the repeated immunization of animals and the use of highly specialized, laborious techniques to produce specific polyclonal sera or mAbs with high specificity and affinity. These limitations have generated the need to investigate potential artificial recognition sites [18]. Among artificial receptors, molecularly imprinted polymers have a proven potential as synthetic receptors in numerous applications, ranging from chromatography to sensor fabrication [18–21].

In this study, the feasibility of applying the molecular imprinting technique to the detection of DON using a surface plasmon resonance (SPR) transducer was investigated. For this aim, DON-imprinted polypyrrole (DON-MIPPy) films were fabricated on a SPR sensor chip and their surface characteristics and DON-binding properties were examined.

## 2. Materials and Methods

### 2.1. Reagents and Materials

DON, 3-acetyldeoxynivalenol (3α-acetoxy-7α,15-dihydroxy-12,13-epoxytrichothec-9-en-8-one, 3-ADON), 15-acetyldeoxynivalenol (15α-acetoxy-7α,15-dihydroxy-12,13-epoxytrichothec-9-en-8-one, 15-ADON), pyrrole, tetraethylammonium tetrafluoroborate and acetonitrile were obtained from Sigma-Aldrich (St. Louis, MO, USA). Pyrrole was purified by distillation, and then stored at 4 °C in the dark. Tetraethylammonium tetrafluoroborate (99%) and acetonitrile (anhydrous grade, 99.8%) were used without further purification. Other chemical reagents, such as methanol and chloroform, were of certified analytical grade and were used without further purification.

### 2.2. Instruments

The electropolymerization of pyrrole was performed using a CHI 1230B electrochemical analyzer (CH Instruments Inc., Austin, TX, USA). The MIPPy film was characterized via atomic force microscopy (AFM) using an XE-150 system (Park Systems Corp., Suwon, Korea) and via SPR. The reactions between DON and the MIPPy film were measured using a homemade SPR, consisting of a light source, an integrated flow cell, an interface controller, and application software [21–23].

### 2.3. Preparation of MIPPy and Non-MIPPy Film

Electropolymerization of pyrrole was carried out using a three-electrode electrochemical system. An Au film (50 nm thickness) was deposited on a glass slide (18 × 18 mm^2^) via electron-beam evaporation. This film served as a cathode after making contact with the aluminum foil in the Teflon electrochemical cell, using Ag/AgCl as a reference electrode and a platinum grid as a counter electrode [21]. For MIPPy preparation, 1 mL of an acetonitrile solution containing 0.5 M pyrrole as a functional monomer, 0.2 M tetraethylammonium tetrafluoroborate as an electrolyte, and 3.0 mM DON template was placed on the Au surface to cover the counting and reference electrodes with a constant potential of 0.9 V and a current density of 5 mC/cm^2^ at room temperature. After the synthesis, the films were subjected to a successive washing procedure using acetonitrile, methanol, and chloroform to remove any DON and electrolytes entrapped in the polymeric matrix. Subsequently, the films were dried using N_2_ gas after overnight storage in methanol. The non-MIPPy film was prepared according to the procedure described above in the absence of the template molecule.

### 2.4. Characterization of the MIPPy Film

The thickness of the MIPPy film and the changes in surface morphology were characterized using AFM. To evaluate changes in surface morphology and film thickness, AFM images produced with an XE-150 microscope were acquired using a noncontact mode with 910-NCHR silicon cantilevers (Nanosensors, Neuchatel, Switzerland) at a resonance frequency of 300–340 kHz. Scanning parameters were adjusted to provide clear images. Most of the images were acquired in the 1 × 1 μm^2^ range with 512 × 512 pixels. The reaction between DON and MIPPy was measured using a SPR. Au-coated glass substrates were index matched using a BK7 prism (n = 1.515, Sigma-Aldrich). Optical contact between the prism and the Au substrate was achieved using a refractive index-matching fluid (nD = 1.515–1.517) (Merck, San Diego, CA, USA). A *p*-polarized He–Ne laser set at 670 nm was used as the probe beam. The intensity of the beam reflected through the prism was measured using a S1337 photodiode detector (Hamamatsu Photonics, Hamamatsu, Japan) [21–23]. The incident angle of the prism was changed using a D80 motorized rotary stage and its controller (Suruga Seiki, Shizuoka, Japan), with a minimum resolution of 0.004°.

### 2.5. SPR Measurements

To obtain SPR measurements, MIPPy-coated Au chips were mounted on the SPR cell, as described in a previous report [21]. Evaluation of the optical parameters of MIPPy chips was performed under the following conditions: the incident angle of the laser varied from 35 to 55°, with a resolution of 0.04° in air. The reflected intensity of the SPR, which was used to investigate the recognition between DON and the MIPPy (or non-MIPPy) film, was measured according to the incident angle of the laser (from 68 to 88°), using a resolution of 0.012° and intervals of 10 min at room temperature. The resonance angle of the SPR, which was determined at the minimum intensity of the SPR curve, was compared between distilled water without DON and a standard solution of DON (0.1–100 ng/mL) and plotted against time of measurement. To evaluate the cross-selectivity of MIPPy films, two acetylated analogs were injected onto the MIPPy film and shifts in their resonance angles were compared with that induced by DON at a concentration of 10 ng/mL.

## 3. Results and Discussion

### 3.1. Characterization of the MIPPy film

**Figure 1.**
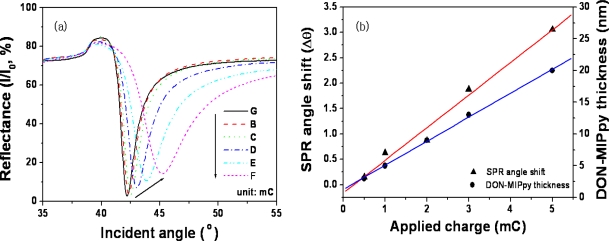
SPR spectra (a) and thickness change (b) during the formation of the DON–MIPPy film according to the charge applied at 900 mV on bare Au. (**a**) Resonance angle curve of the dried films measured in air. (**b**) The left-side Y-axis (▴) represents the difference of the resonance angle of the MIPPy film (θ) and bare (θ_bare_) obtained from Figure 1(a), and the right-side Y-axis (•) represents the film thickness, as assessed using AFM.

Because SPR signals depend on the surface properties of the film, the MIPPy film formed should have a thickness and roughness that are adequate for the detection of DON using SPR [21,24,25]. Figure 1(a) shows a plot of the reflectance *vs* incident angle depending on the charge applied at 900 mV on bare Au. The resonance angle of bare Au (42.20°) increased as a result of the increase in electric charge, because of the change in MIPPy film thickness [22,26,27]. Figure 1(b) shows the response of the SPR sensor and the thickness of DON–MIPPy film for the various electric charges applied. The SPR responses increased in proportion to the electric charges applied, because of the change in refractive index near the SPR chip. A plot of the changes in resonance angle *vs* electric charge exhibited linearity from 0.5 to 5.0 mC for DON–MIPPy SPR chips. A thickness of 3–27 nm, as measured using AFM, was observed for DON–MIPPy films in cases of application of 0.5–5.0 mC electric charges. In particular, an electric charge of 1.0 mC yielded a DON–MIPPy film thickness of ∼6 nm.

**Figure 2.**
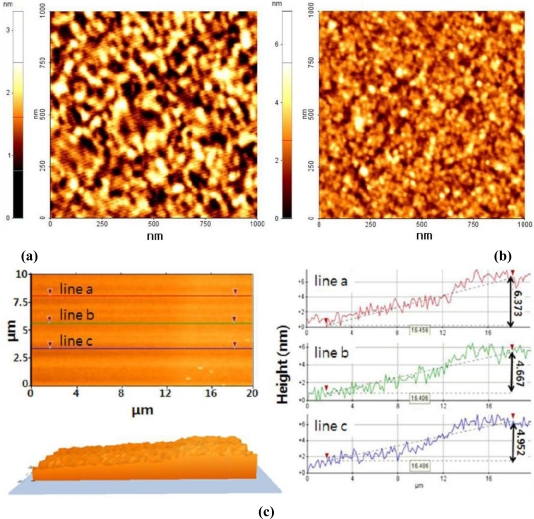
AFM images of surface morphology changes before (a) and after (b) the formation of the DON–MIPPy film. **(a)** is the surface image of the bare Au chip used for SPR measurements and **(b)** is the image of the DON–MIPPy film (1 mC; 900 mV) formed on the bare Au chip, and **(c)** is the image (2D, 3D and line profile) scanned on the border line between Au and MIPPy film.

The surface of DON–MIPPy films (area, 1 × 1 μm^2^) was imaged using AFM in a noncontact mode. Figure 2(a) shows that bare Au exhibited polycrystalline morphology with visible particle boundaries. After deposition (1 mC) of the MIPPy film, the phase contrast and the surface roughness were greatly changed, indicating the presence of the film layer on the Au surface. The mean roughness and mean value of the surface relative to the center plane of bare Au and MIPPy were estimated at 0.35 and 0.58 nm, respectively. The highest peak in the measuring region, which represents the MIPPy maximum thickness, and the mean difference between the peak and the valley at each of five positions, which represents the thickness deviation, were measured as 7.17 and 1.08 nm, respectively. Additionally, in order to measure the thickness of MIPPy film on Au surface, 20 × 10 μm^2^ of film area was scanned. Figure 2(c) was an AFM image of the border between bare Au (left side) and MIPPy films (right side). The line profiles (line a, line b and line c) of AFM image indicate the film thickness from bare Au as 6.373, 4.667, and 4.952 nm. Overall, these results imply that MIPPy films were formed on the Au surface with a thickness of 5∼6 nm.

### 3.2. Binding Properties of DON to the MIPPy Film

**Figure 3.**
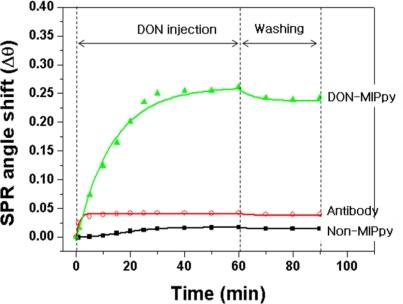
Reaction sensorgram of the DON–MIPPy (▴), DON–antibody (○), and non-MIPPy (▪) films (used as receptors) formed on SPR bare Au chips using the DON solution (100 ng/mL). The SPR angle shift represents the difference between the angle after injection of the DON solution and the angle of the running solution.

The performance of the DON–MIPPy film prepared on the bare Au chip was assessed in comparison with nonimprinted polypyrrole (non-MIPPy) and anti-DON antibody, which were used as sensing receptors, via SPR measurements. Figure 3 shows an SPR sensorgram obtained after injection of DON solution as an analyte onto the DON–MIPPy, non-MIPPy, and anti-DON-antibody-immobilized SPR sensor chips. The DON–MIPPy sensor chip exhibited a larger shift in resonance angle compared with the non-MIPPy and anti-DON-antibody-immobilized sensor chips. The resonance angle of the DON–MIPPy sensor chip shifted by 0.26° (range, 84.28–84.54°) after injection of 100 ng/mL of DON into the SPR cell, and saturation was observed after 40 min. Washing of the MIPPy film using an eluent (distilled water) led to a shift in SPR angle of 0.24°compared with that observed before injection of the DON solution, which indicates that the cavities of DON were well formed. In contrast, the anti-DON-antibody-immobilized SPR did not detect DON. We do not have a clear explanation for this finding; however, differences in the number of binding sites for DON, and in the affinity of the anti-DON antibody might be related to the observed difference in sensitivity. The resonance angle of the non-MIPPy film without DON binding sites shifted by as little as 0.001° and this minimal shift resulted from weak and nonspecific adsorption caused by the random arrangement of the functional groups in the non-MIPPy film. This result was similar to that reported in a previous study, in which zearalenone and caffeine were detected using a non-MIPPy SPR and quartz crystal microbalance (QCM), respectively [21,28].

**Figure 4.**
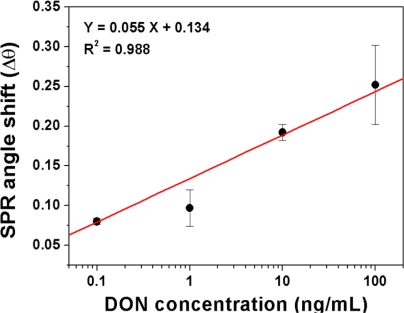
Resonance angle shift of DON–MIPPy chips according to varying concentrations of the DON solution. For DON measurement, at least, three different replications were done.

The effect of various concentrations of DON on the resonance angle shift of DON–MIPPy sensor chips is shown in Figure 4. The plot of the resonance angle shift versus DON concentration showed good linearity at DON concentrations ranging from 0.1 to 100 ng/mL (R^2^ = 0.988). This result suggests that molecular imprinting can be applied to the detection of DON.

**Figure 5.**
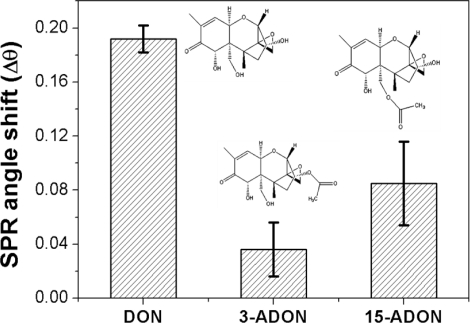
Selectivity of DON–MIPPy chips regarding several structurally similar DON analogs. The SPR angle shift represents the difference in resonance angle before and after the addition of 10 ng/mL of DON or its analogs onto the DON–MIPPy chip.

To evaluate the cross-selectivity of the MIPPy film, changes in the resonance angle of DON were compared with those of other structurally related analogs (Figure 5), such as 3-ADON and 15-ADON. The selectivity efficiency of the DON–MIPPy film was defined using the following equation:

(1) $$\text{Selectivity efficiency }\left(\%\right)=\frac{\Delta R_{\text{analogues}}}{\Delta R_{\text{Deoxynivalenol}}}\times 100$$

where ΔR is the difference in SPR resonance angle before and after addition of the DON analogs. According to Equation (1), the selectivity efficiency of DON, 3-ADON, and 15-ADON was calculated as 100, 19, and 44%, respectively. These results imply that MIPPy films exhibit high selectivity for DON. The imprinting of DON in the polypyrrole (Ppy) matrix is facilitated by the formation of a hydrogen bond between the functional groups of DON and the imine hydrogen atoms in the pyrrole molecule [29,30].

The DON molecule contains six oxygen atoms consisting of three alcoholic OH groups, a carbonyl group, a cyclic ether group, and an epoxide group, which allow for multiple hydrogen bonds in the Ppy matrix. Cross-reactivity results showed that the presence of free hydroxyl groups at positions 3 and 15 of the 8-ketotrichothecene moiety appears to be favorable to the determination of selectivity [31]. The comparison of selectivity efficiency between 3-ADON and 15-ADON revealed that the selectivity efficiency of 15-ADON was 2-fold that of 3-ADON; these two molecules differ only in the position of a hydroxyl group and an acetyl ester group on the 8-ketotrichothecene structure. This implies that the free hydroxyl group located at position 3 of DON may be the most influential recognition point of the functional monomer, pyrrole. These selectivity values differed from previous reports that used anti-DON polyclonal or monoclonal antibodies. The anti-DON antibody recognizes 3-ADON, 15-ADON, and DON well. Various approaches were used to show that the cross-reactivity to 15-ADON was negligible, whereas that to 3-ADON varied from <100% to >300%, depending on the antibody used [32–34]. We do not have an explanation for this discrepancy; however, differences between the recognition sites generated during antibody production and the imprinting of DON on the Ppy matrix might be related to the differences in selectivity observed. In general, the affinity and selectivity of the antibody depended on the type of carrier protein that was coupled to DON prior to immunization (e.g., human serum albumin, keyhole limpet hemocyanin, or ovalbumin A) and on the DON protein-binding location used for antibody production. Regarding the potential of the two acetylated DON analogs to contribute to the overestimation of results, their contribution to DON concentration is, in most cases, insignificant, because of the relatively low levels of 3-ADON and 15-ADON occurring commonly in cereals [35,36].

## 4. Conclusions

In this study, the MIP technique, which functions as a synthetic recognition element, was combined with an SPR transducer to detect the mycotoxin DON. The polymer was prepared via electropolymerization of pyrrole onto a bare Au SPR chip in the presence of a template DON molecule. The successful synthesis of the MIPPy film on bare Au was demonstrated by determining the characteristics of the film (thickness and surface morphology) using SPR and AFM. The MIPPy–SPR sensor exhibited a linear response for the detection of DON in the range of 0.1–100 ng/mL (R^2^ = 0.988), with a detection limit of >1 ng/mL. The selectivity efficiency of DON and of its acetylated analogs (3-ADON and 15-ADON) was 100, 19, and 44%, respectively, indicating the high binding affinity for DON. These results suggest that a combination of SPR sensing with MIPPy film is a potential method for the detection of DON. However, for practical applications, this approach requires optimization and validation using natural samples of complex composition.
